# Comparative Analysis of Biofilm Removal Efficacy by Multisonic Ultracleaning System and Passive Ultrasonic Activation

**DOI:** 10.3390/ma12213492

**Published:** 2019-10-25

**Authors:** Hae Won Choi, Seong Yeon Park, Mo Kwan Kang, Won Jun Shon

**Affiliations:** 1 Department of Dental Biomaterials Science, School of Dentistry, Seoul National University and Department of Orthodontics, Institute of Oral Health Science, Samsung Medical Center, Sungkyunkwan University School of Medicine, Seoul 03080, Korea; orthochoi7@gmail.com; 2 Dental Research Institute and School of Dentistry, Conservative Dentistry, Seoul National University, Seoul 03080, Korea; alpharigel@hanmail.net; 3 Department of Endodontics, University of California, Los Angeles, CA 10833, USA; mkang@dentistry.ucla.edu

**Keywords:** *Enterococcus faecalis* biofilm, GentleWave System, passive ultrasonic activation, root canal disinfection

## Abstract

The purpose of this study was to compare disinfection and the biofilm removal efficacy of the GentleWave System (Sonendo, Inc., Laguna Hills, CA, USA) with passive ultrasonic activation method. Forty-seven freshly extracted human molars were inoculated with *Enterococcus faecalis* and cultured for five weeks to establish biofilm. Eight molars were tested for confirmation of infection. Four of the eight teeth were not inoculated in order to provide a negative control. The remaining 39 inoculated molars were randomly separated into three treatment groups (n = 13 per group): Group 1—no treatment, Group 2—conventional rotary instrumentation and passive ultrasonic activation, and Group 3—minimal instrumentation and the GentleWave System treatment. Roots were subsequently prepared per standard histological tissue processing procedures. Modified Brown and Brenn stained sections and Hematoxylin and Eosin stained sections were visualized at 4× and 13.5× magnification using a stereomicroscope. The sections were scored and blindly analyzed by two independent evaluators, including a histopathologist, to evaluate the presence of biofilm on canal wall. A significant difference was found between Group 2 and Group 3 in both apical and middle regions (*p* = 0.001) of the mesial roots of mandibular molars and mesiobuccal roots of maxillary molars. Group 3 revealed significantly less biofilm than the controls (*p* = 0.003). The GentleWave System demonstrated significantly greater reduction in biofilm within the mesial roots of mandibular molars and mesiobuccal roots of maxillary molars than those treated with conventional rotary instrumentation and passive ultrasonic activation protocol.

## 1. Introduction

It has been well established that endodontic disease is a biofilm-mediated infection. Endodontic pathogens often form a biofilm where bacterial populations are enclosed in a three-dimensional polysaccharide matrix, the result of which is a highly resistant and adherent infectious community [1,2]. Therefore, the elimination of bacterial biofilms is an essential element for the successful outcome of endodontic treatment.

One facultative anaerobic species, *Enterococcus faecalis* (*E. faecalis*), is particularly predominant in endodontic infections because of its ability to suppress lymphocyte action, its ability to prevail under severe environmental conditions, and its resistance to antibodies and antimicrobials [1,3]

The complex anatomy of the root canal system makes complete removal of bacterial biofilms challenging. Conventionally, a chemo-mechanical approach is used to disinfect the root canal system. This method relies heavily on mechanical instrumentation to debride the pulpal tissues and biofilm from the main canal space followed by antimicrobial irrigants to disinfect bacteria and dissolve tissues. However, multiple in vivo and in vitro studies have shown the presence of residual bacteria in more than 80% of all cases, particularly in the apical region, when treated with the conventional methods [2,4,5,6]. Another more recent methodology used in the disinfection of endodontic biofilms is ultrasonic activation. Previous studies have shown that activation of antimicrobial irrigants with ultrasonic energy increases their antibacterial effects when compared to conventional mechanical instrumentation and irrigation [7]. Nonetheless, currently available ultrasonic technologies cannot completely remove bacterial biofilm from infected root canals [8,9,10]. Recently, in vitro studies have shown that the GentleWave System (Sonendo, Inc., Laguna Hills, CA, USA) allows for higher tissue dissolution rate, better removal of pulp tissue remnants, and complete removal of calcium hydroxide from apical and lateral canals into tubules [11,12,13,14]. The novel GentleWave (GW) system (Sonendo Inc., Laguna Hills, CA, USA) continuously supplies a degassing microbubble solution throughout the root canal system via a handpiece positioned on an accessed occlusal tooth surface area. The handpiece delivers a stream of irrigant fluid into the pulp chamber, and the built-in suction removes the outflowing fluid, creating negative pressure within the root canal system. 

However, there are no studies to date assessing the ability of the GentleWave System on the removal of biofilm from the canal wall. Thus, the purpose of this study is to assess the ability of the GentleWave System compared to passive ultrasonic activation on the removal of biofilm in complex anatomical situations (mesiobuccal roots of maxillary molars and mesial roots of mandibular molars).

## 2. Materials and Methods

### 2.1. Sample Selection

This study was reviewed and approved by the Institutional Review Board of Seoul National University Hospital (IRB No. S-D20160011). Forty-seven recently extracted human first or second molars (both mandibular and maxillary) were collected and stored in 1X phosphate buffered solution (PBS) at 4 °C until use. The teeth were radiographically assessed and those that met the exclusion criteria were not utilized. Briefly, any teeth with decay or fractures below the cemento-enamel junction (CEJ), internal or external root resorption, open apices, or previous root canal therapy were excluded.

### 2.2. Sample Preparation

When present, caries were removed and missing coronal tooth structure was restored using etchant (Etch-Rite, Pulpdent, Watertown, MA, USA), bonding agent (Optibond, Kerr, Orange, CA, USA), and Virtuoso^®^ flowable light-cure composite (Denmat, Lompoc, CA, USA). Following endodontic access, all teeth were firmly secured and sealed within a water-saturated porous medium using an adhesive (McMaster-Carr, Los Angeles, CA, USA) to simulate blood-saturated periapical tissue [14]. Reproducible glide paths and working length were established using a #10 K-file (MANI K-files, Utsunomiya, Japan). The working length was defined as 1 mm from the apical foramen. Prior to inoculation, root canals were minimally shaped up to #15/.02 (MANI K-files, Utsunomiya, Japan). One mL of saline was delivered in between each instrument using a syringe and 30G Max-i-Probe needle (Dentsply Rinn, Elgin, IL, USA) to flush out the dentin debris created during instrumentation. The instrumented molars were treated with the GentleWave System using 3% NaOCl and 8% Ethylene diamine tetra acetic acid (EDTA), as described previously [14]. The samples were subsequently placed in glass vials with sterile PBS and autoclaved prior to incubation with *E. Faecalis*.

### 2.3. Sample Inoculation

Forty-seven molars were placed individually in 15 mL centrifuge tubes. Four molars were injected with 120 µL brain heart infusion media (BHI, Teknova, Hollister, CA, USA) using 30G Max-i-Probe needle. The molars were incubated in fresh 5 mL of BHI. The tubes were securely closed and centrifuged at 1150 ×g for 5 min. This process was repeated 3 times. The four molars served as negative controls for testing the confirmation of infection. 

For the remaining 43 molars, 120 µL of *E. faecalis* (ATCC#19433, Manassas, VA, USA) in BHI was injected into the roots. The purity and identity of the strain was confirmed, as described previously [9]. Five mL of BHI was carefully pipetted into the centrifuge tube in the area surrounding the tooth. The tubes were securely closed and centrifuged at 1150 ×g for 5 min. This process was repeated 3 times. The implemented culturing method was modified to the one described previously [12]. Four teeth served as positive controls for the purpose of confirming the infection. The centrifuged tubes were filled with an additional 5 mL of BHI and incubated for 5 weeks at 37 °C. BHI was replenished biweekly.

### 2.4. Confirmation of Infection 

The infection in the root canal system was confirmed by testing for turbidity within the media in contact with the apical portion of the root. Additionally, the biofilm growth was confirmed by imaging the 4 negative and 4 positive controls: 4 molars (2 negative and 2 positive controls) one day after inoculation, and 4 molars (2 negative and 2 positive controls) after the 5-week inoculation period. Images were acquired using scanning electron microscopy (SEM) and the biofilm growth was confirmed. In short, the samples were fixed overnight in 4% buffered paraformaldehyde. The roots were separated, split longitudinally, and dehydrated with graded alcohol. The dehydrated samples were sputter coated with gold/palladium and examined under low vacuum SEM (TM3000, Hitachi, Tokyo, Japan) at a magnification of 600×. The coated root samples were carefully examined along the canal and especially the apical thirds. 

### 2.5. Treatment Groups

The remaining 39 teeth were randomly divided into the three groups, ensuring that each group had an equal amount of maxillary molars (5 per group). The average length of the 39 roots was 10.1 mm ± 1.03 mm. For this study, only roots with more complex anatomies, i.e., mesiobuccal roots of maxillary molars and mesial roots of mandibular molars were evaluated [15,16].

**Figure 1:**
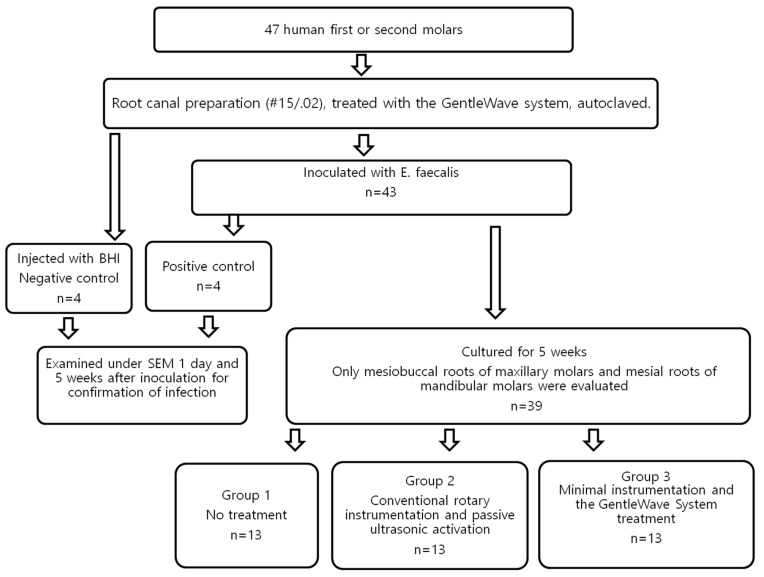
Flow diagram of all the treatment procedures.

(1) Group 1—No treatment (n = 13): Teeth in the control group did not undergo any endodontic treatment after inoculation with *E. faecalis*.
(2) Group 2—Conventional rotary instrumentation and passive ultrasonic activation with the PiezonMaster™ 700 ESI tip (n = 13): The canals were instrumented to a size #20 K-file to reach the working length. The canals were further instrumented with Protaper rotary filing system (Protaper, Irvine, CA, USA) in a sequence of S1/S2/F1/F2 followed by #35/.04 EndoSequence rotary file (Brasseler, Savannah, GA, USA) to working length. 1 mL of 3% Sodium hypochlorite (NaOCl) was again delivered in between each instrument using a syringe and 30G Max-i-Probe needle to flush out the dentin debris created during instrumentation. The canals were irrigated with 3% NaOCl at a flow rate of 3 mL/min for 10 s per canal. Each canal was activated for 60 s per canal at maximum power in Endo mode using PiezonMaster 700 with an ESI tip (Electro Medical Systems, Nyon, Switzerland) [15,17,18,19]. This irrigation-activation process was repeated once for all the canals at the end of root canal instrumentation. The canals were then irrigated with sterile distilled water using a Max-i-Probe needle for 10 s per canal. This was followed by irrigation with 17% EDTA for 10 s per canal at a flow rate of 3 mL/min. The needle was placed 2 mm from the apical foramen. Final activation was performed with sterile distilled water for 10 s per canal. All canals were dried with sterile paper points.
(3) Group 3—Minimal instrumentation and the GentleWave treatment (n = 13): The canals were instrumented with #15/.04 EndoSequence rotary files. 1 mL of saline was delivered in between each instrument using a syringe and 30G Max-i-Probe needle to flush out the dentin debris created during instrumentation. For the GentleWave treatments, the handpiece tip was placed inside the pulp chamber of the accessed tooth. According to the manufacturer’s instruction for use, the treatment consisted of 3% NaOCl for 5 min, sterile-distilled water for 30 s, 8% EDTA for 2 min, and sterile-distilled water for 15 s, sequentially [11,12,13,14,20]. All the canals were dried with sterile paper points. Only 8% EDTA was used, as recommended by the manufacturer. All the treatment flows are illustrated in Figure 1.

### 2.6. Sample Processing

Each canal was irrigated and filled overnight with 1 mL of 10% buffered formalin to allow for fixation of any remaining biofilm, as described earlier [14]. Roots were embedded in paraffin. 

The roots were divided into three regions as—apical region (with sub-regions at 1, 2, and 3 mm) and middle region (with sub-regions at 4, 5, and 6 mm). Four slices at 1 mm increments were obtained at each sub-region using a rotary microtome (Leica RM2235, Leica, Wetzlar, Germany) for a total of 24 sections per root. 12 sections were stained using hematoxylin and eosin (H&E) and 12 sections were stained using modified Brown and Brenn (B&B). The H&E stained and B&B stained cross-sections were further examined using an optical stereomicroscope (Leica DMLB, Leica, Wetzlar, Germany) and images were acquired using a camera (Leica DFC290, Leica, Wetzlar, Germany). For further analysis, B&B stained slides were examined at 13.5× magnification. Resulting images were subjected to morphometric analysis [21]. 

### 2.7. Data Analysis

Approximately 936 sections (39 molars × 24 sections/root) stained with H&E or modified B&B were evaluated for the three treatment groups. For quantification purposes, a modified four-score scale system was utilized [2]. The accuracy of the bacterial staining method was tested [22]. The canals and the corresponding sections of each root were thoroughly examined by two independent, trained, and blinded evaluators, including an experienced histopathologist. Presence of bacterial biofilm on the main canal (middle) with isthmus region and apical root canal surface was confirmed by evaluating both H&E and B&B stained specimens. The level of biofilm attachment under histologic examination was scored according to the following criteria: Score 0—no biofilm; Score 1—sparse and detached bacteria; Score 2—well-defined biofilm, small colonies (<50% of the canal wall covered); and Score 3—well-defined biofilm, large colonies (>50% of the canal wall covered). The presence biofilm scores were compared between experimental groups and between canals and isthmus.

### 2.8. Statistical Analysis

Statistical analysis was performed using the non-parametric Kruskal–Wallis test with Dunn’s multiple comparisons, and Mann–Whitney tests (*p* < 0.05) with Prism 5.0 (GraphPad Software Inc., La Jolla, CA, USA) analytical tool. Cohen’s Kappa values were calculated to note the differences in the evaluators. A priori power analysis was performed using GPower (Version 3.1.9.2, Universität Kiel, Germany) to ensure that the results attained adequate power [23]. When a total of 39 teeth were used, the power (1-β) was equal to 0.99 when α = 0.05, and analysis parameters were set to a one-tailed *t*-test.

## 3. Results

**Figure 2:**
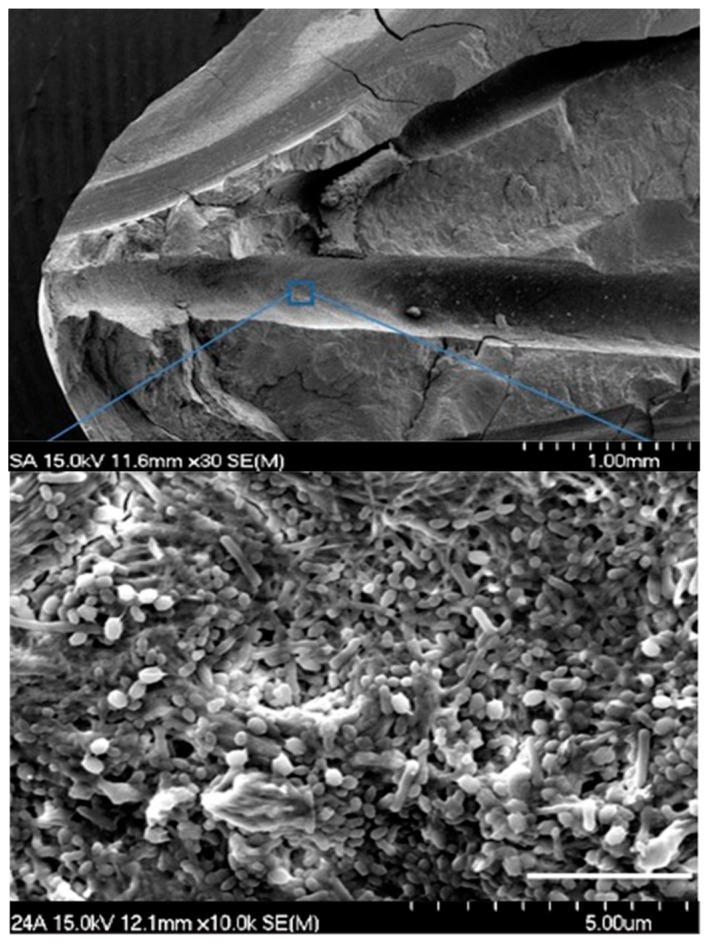
Representative SEM images of the apical-third region from the molars inoculated with *E. faecalis* (Upper, 30× magnification), randomly selected with apical-third region (Lower, 10,000× magnification) in positive Control group.

**Figure 3:**
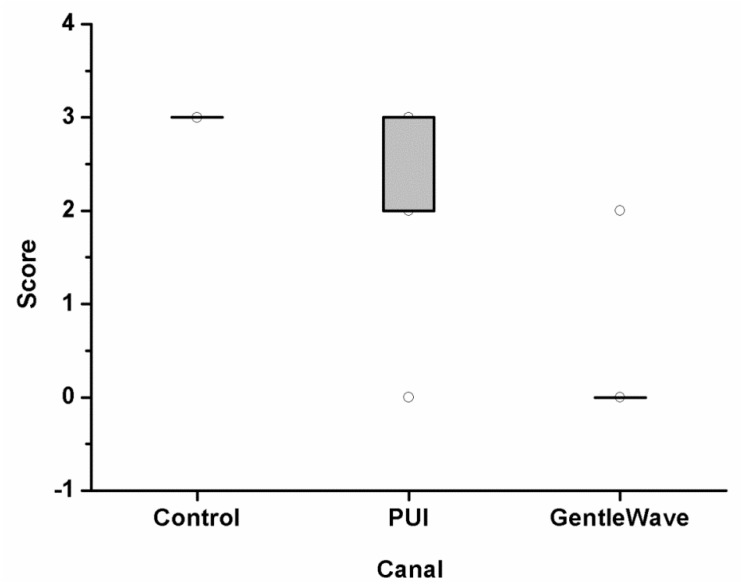
The level of biofilm attachment scores from three independent evaluators for 39 molars in the main canal (middle) region. The samples were either untreated (Group 1), treated with passive ultrasonic activation system (Group 2), or treated with the GentleWave system (Group 3). The modified scores utilized were—Score 0: no bacteria; Score 1: sparse and detached bacteria; Score 2: well defined biofilm (small colonies, <50% of the canal wall covered); and Score 3: well defined biofilm (large colonies, >50% of the canal wall covered). The average scores analyzed for the canals between Group 2 and Group 3 were statistically different (*p* < 0.001).

**Figure 4:**
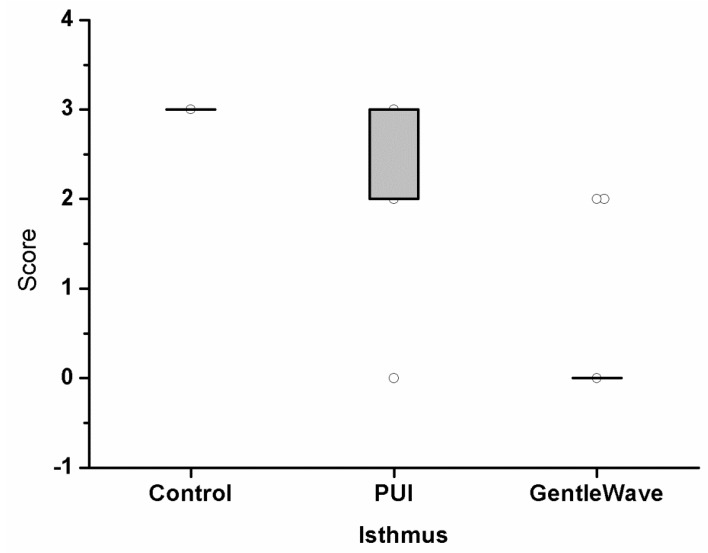
The level of biofilm attachment scores from three independent evaluators for 39 molars in the isthmus region. The samples were either untreated (Group 1), treated with passive ultrasonic activation system (Group 2), or treated with the GentleWave system (Group 3). The modified scores utilized were—Score 0: no bacteria; Score 1: sparse and detached bacteria; Score 2: well defined biofilm (small colonies, <50% of the canal wall covered); and Score 3: well defined biofilm (large colonies, >50% of the canal wall covered). The average scores analyzed for the canals between Group 2 and Group 3 were statistically different *(p* < 0.001).

Images of the positive control group were acquired using scanning electron microscopy (SEM), and the biofilm growth was confirmed in Figure 2. Upon evaluation of the histologic specimens for the level of existing biofilm on the canal wall, the scores from the two independent evaluators were compiled together. In occasions of disagreements, the images were reanalyzed and discussed to obtain a consensus. The levels of biofilm attachment score are shown in Table 1, Figure 3 and Figure 4 for both the main canal (middle) and the isthmus regions. The kappa coefficient was 0.76 and 0.73 showing a strong agreement between the examiners. The median scores of the main canal region in Group 1, Group 2 and Group 3 are 3, 2 and 0 respectively (A). The median scores of the isthmus region in Group 1, Group 2 and Group 3 are 3 and 0 respectively (B).

**Figure 5:**
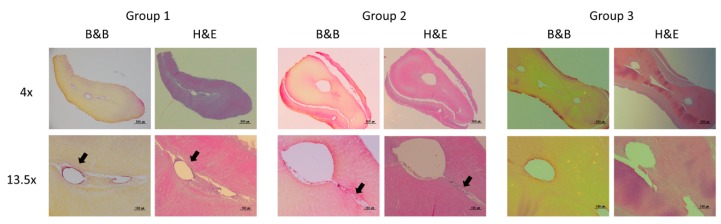
Representative histological images of the apical-third region from the molars left untreated (Group 1), treated with passive ultrasonic activation system (Group 2), or treated with the GentleWave System (Group 3). Note the well defined bacterial biofilm in contact with the root canal wall in Group 1 and 2 compared to Group 3.

Histological images of representative cross-sections in the apical-third regions are presented in Figure 5. The presence of the biofilm was observed in the canal space for Group 1. Even though there was a reduction in the amount of biofilm in Group 2, the presence of a persistent biofilm could still be visualized. In Group 3, a clean and disinfected canal space was observed. 

**Figure 6:**
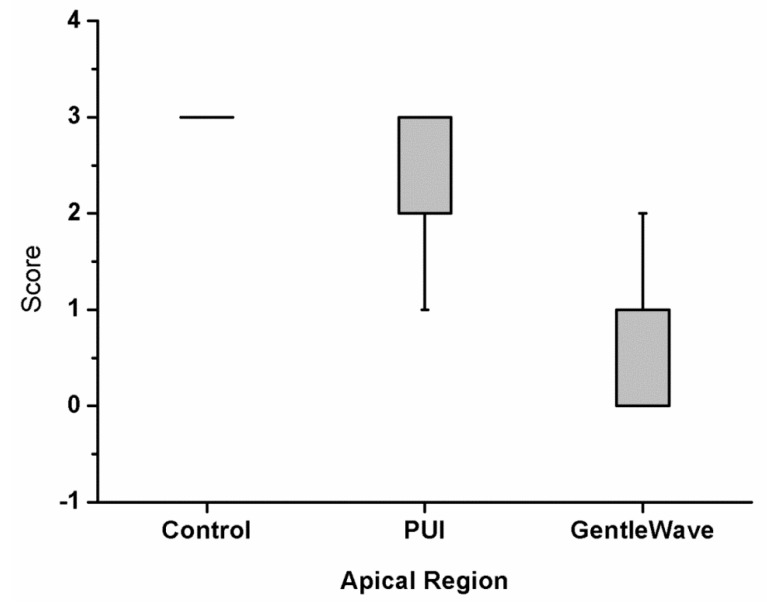
The level of biofilm attachment scores from all the apical sections and the two independent evaluators for the 39 molars left untreated (Group 1) or treated with passive ultrasonic activation (Group 2) or treated with the GentleWave System (Group 3). The modified scores utilized were—Score 0: no bacteria; Score 1: sparse and detached bacteria; Score 2: well defined biofilm (small colonies, <50% of the canal wall covered); and Score 3: well defined biofilm (large colonies, >50% of the canal wall covered). The average scores analyzed for the canals between Group 2 and Group 3 were statistically different (*p* < 0.001).

The results of the analysis of biofilm removal from the apical-third regions are summarized in Figure 6. Group 1 contained large biofilm along the canal wall and inside the main canal. Group 3 had the lowest scores compared with Group 2 and Group 1. In the main canal and isthmus regions, the differences between Group 3 and Group 2 was statistically significant (*p* = 0.003 and *p* < 0.0001, respectively). The results of this study indicate that Group 3 disinfects significantly greater than Group 2. 

## 4. Discussion

The most comprehensive explanation for endodontic failures is the prevalence of bacteria in the root canal space. Although mechanical preparation of the infected root canal system reduces the biofilm load, it is unreliable in rendering the canals biofilm-free [24,25]. Numerous antibacterial solutions have been developed and utilized in recent years [26,27]. However, microbial communities established in biofilms are difficult to eradicate, and the bacteria encapsulated in mature biofilms can be highly resistant to antimicrobial therapies [28]. This study showed that the amount of infected biofilms removed in the apical and isthmus regions of root canal systems was greater with the GentleWave System than with passive ultrasonic activation. 

The effectiveness of passive ultrasonic activation on intra-radicular *E. faecalis* biofilms was previously studied [29]. However, these studies were performed on 3 day old immature biofilms and in single-rooted teeth. The authors utilized the non-specific SEM method to analyze the results. 

On the contrary, in the present study, five-week old mature biofilms in molars were used to compare the disinfection efficiency of the GentleWave System and the passive ultrasonic irrigation. A possible reason for the observed cleanliness of the root canal system using the GentleWave System might be the underlying technology and the effective and continuous flow of treatment fluids: NaOCl-water-EDTA-water [13]. The notable finding of this study is that the residual biofilm removal effect of Group 3 is superior to Group 2 in the isthmus and apical region, which is known to have complex root canal anatomy. This result can be deduced from the unique GentleWave System which has the interplay of multisonic energy, vortical fluid dynamics, and chemistry of the treatment fluid, resulting in enhanced dissolution and removal of organic matter, i.e., pulp tissue and the biofilm from the root canal system. When the dynamic irrigant fluid is in contact with stagnant fluids in the pulp chamber, hydrodynamic cavitation caused by sheer force occurs, forming thousands of microbubbles. The bubbles, called cavitation clouds, subsequently implode and create sound waves that cover a broad frequency spectrum that reverberate and contribute to the cleaning throughout the root canal system [11,14]. The presence of multisonic ultracleaning energy in combination with advanced fluid dynamics might have loosened the infected biofilm attached inside the dentinal wall. It is important to note that the treatment tip is positioned inside the pulp chamber and is not required to enter canals or orifices, therefore allowing for minimal instrumentation of the root canals and maintaining the natural tooth structure. However, further understanding of the action of the GentleWave System using flow visualization models are warranted. 

In the current study, it is worth noting that efforts were made to maintain the consistency of the concentration of NaOCl, and the time the canals are exposed to NaOCl, flushed with sterile distilled water, or exposed to EDTA. The times were adjusted to the times indicated, as suggested by the manufacturer recommendations for the GentleWave System. For the passive ultrasonic activation group, full strength EDTA was used to maintain clinical relevancy whereas for the GentleWave System group, only 8% EDTA was used, as recommended by the Instruction for Use (IFU). 

Additionally, the method of inoculation of the molars with the bacteria is a modified method [9,30]. The authors used either single-rooted teeth or dentin samples for their studies, respectively. In the present study, the eight tested molars confirmed infection even in the apical-third regions. Even though the possibility of the biofilm not penetrating deep into the tubules cannot be completely ruled out, it is important to note that the samples were inoculated using the same method for the three treatment groups. Recent study also supported the efficacy of biofilm removal from the infected root canal system by using quantitative real-time Polymerase Chain Reaction (PCR) [31]. The limitations of the culturing technique might underestimate bacterial prevalence and diversity in hostile environments including treated root canals. Oral heterogeneous biofilms can be even more resistant and it is important to utilize multi-species biofilm to understand the disinfection ability of the GentleWave System. Future clinical studies are needed to demonstrate the effectiveness of the GentleWave System in cases of apical periodontitis and downstream periapical healing. 

Under the conditions of the current study, the GentleWave System procedure significantly improved the cleaning of *E. faecalis* biofilm-infected and the disinfection of the root canal system when compared with conventional rotary instrumentation and passive ultrasonic activation. 

## Figures and Tables

**Table 1:** Mean scoring data (Mean N ± SD) evaluated at main canal and isthmus of each group.

Control				PUI				GentleWave				*p*-Value
Canal		Isthmus		Canal		Isthmus		Canal		Isthmus		
Mean	SD	Mean	SD	Mean	SD	Mean	SD	Mean	SD	Mean	SD	
3^w^	0	3^a^	0	1.94^x^	1.20	2.75^b^	0.39	0.15^y^	0.55	0.33^c^	0.74	*p* < 0.05
